# Supplementary material for: Brain-wide neuronal activation and functional connectivity are modulated by prior exposure to repetitive learning episodes
Source: Front Behav Neurosci. 2022 Sep 9;16:907707. doi: 10.3389/fnbeh.2022.907707 (PMC9501867; doi:10.3389/fnbeh.2022.907707)
Supplement: Supplementary file 1 [file Image_1.pdf]

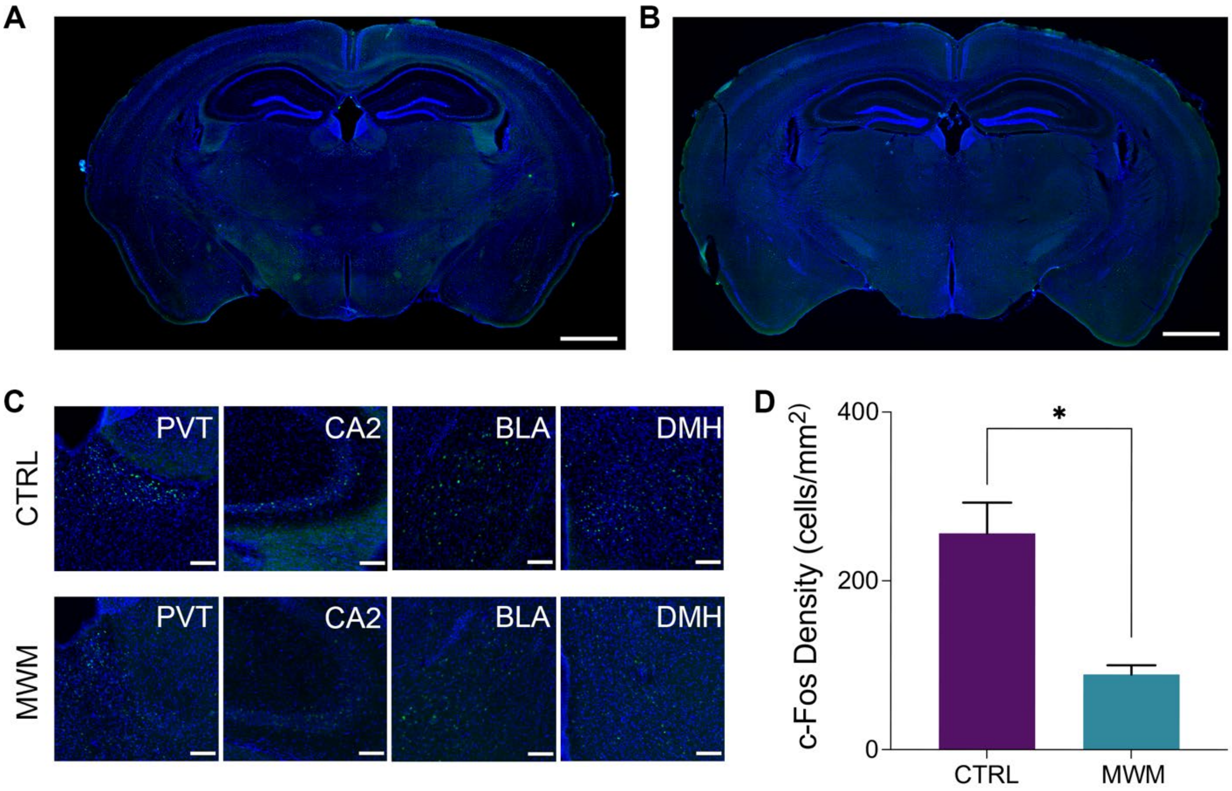

Supplementary Figure S1: Morris water maze training decreased the total brain c-Fos expression density. Representative photomicrographs of c-Fos expression (green) in DAPI-stained (blue) tissue sections from (**A**) control and (**B**) Morris Water Maze trained mice following contextual memory recall. Scale bar represents 1000 μm. (**C**) Photomicrographs highlighting c-Fos expression density in the paraventricular thalamus (PVT), CA2, basolateral amygdala (BLA), and dorsomedial hypothalamus (DMH) Scale bars represent 100 μm. (**D**) The overall c-Fos expression density calculated from across the brain was significantly decreased with MWM training (Two-tailed t test, p=0.0003). Data shown are mean ± SEM.
