# Supplementary material for: Brain-wide neuronal activation and functional connectivity are modulated by prior exposure to repetitive learning episodes
Source: Front Behav Neurosci. 2022 Sep 9;16:907707. doi: 10.3389/fnbeh.2022.907707 (PMC9501867; doi:10.3389/fnbeh.2022.907707)
Supplement: Supplementary file 2 [file Image_2.pdf]

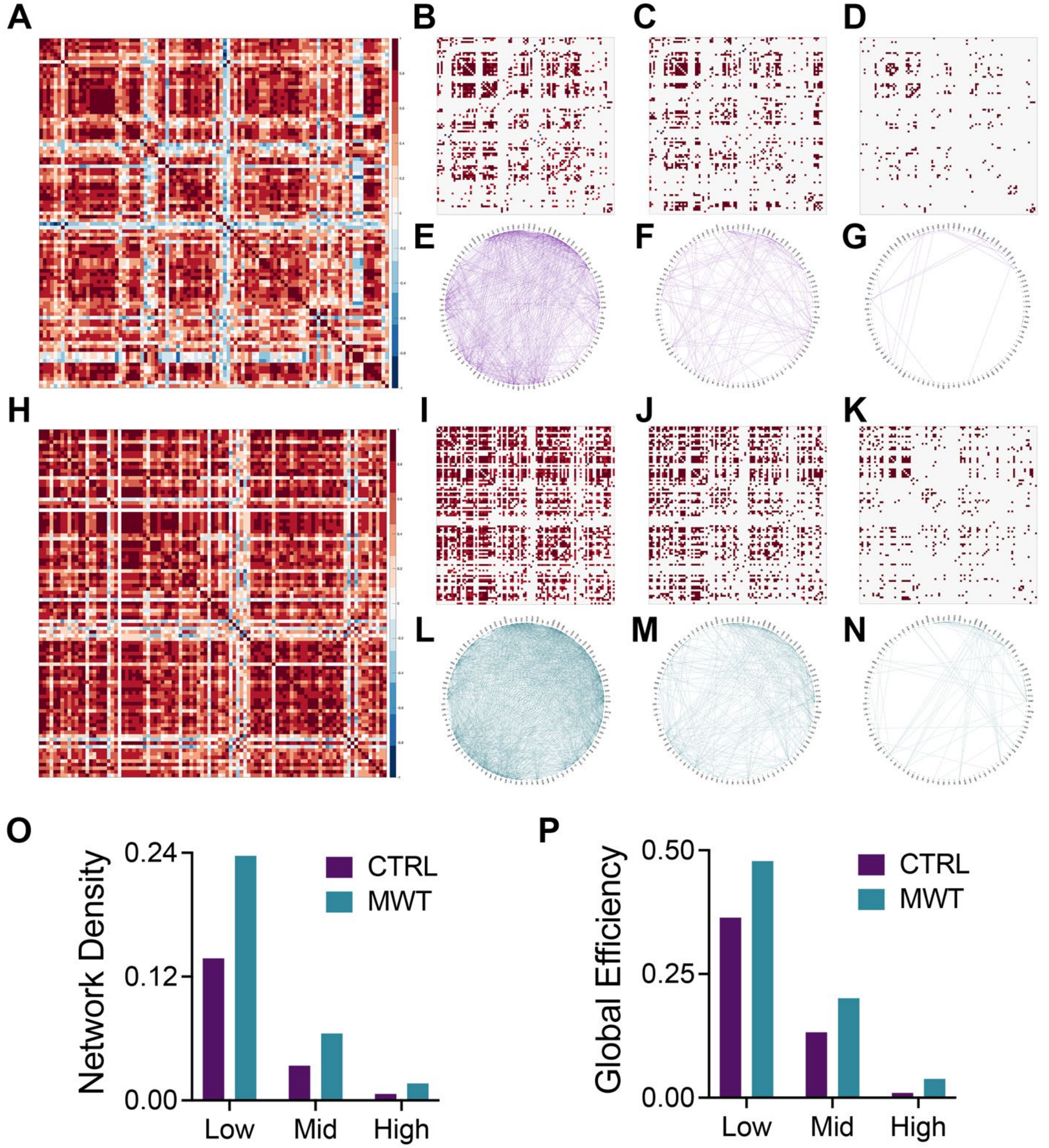

Supplementary Figure S2: Altered global memory network topology induced by Morris water task training is stable across multiple binary network thresholds. Pairwise correlation matrices for and binarized adjacency matrices and circle plots showing significant correlations between regions for control (**A**-**G**) and Morris water task trained (**H**-**N**) groups. Networks were binarized at three different confidence thresholds of $R>0.80$, $P<0.05$ (**B**, **E**, **I**, **L**), $R>0.90$, $P<0.005$ (**C**, **F**, **J**, **M**), and $R>0.95$, $P<0.0005$ (**D**, **G**, **K**, **N**). Across all network thresholds, MWT training increased (**O**) network density and (**P**) global network efficiency. See Online Resource 1 for full list of regions.
