# Supplementary material for: Brain-wide neuronal activation and functional connectivity are modulated by prior exposure to repetitive learning episodes
Source: Front Behav Neurosci. 2022 Sep 9;16:907707. doi: 10.3389/fnbeh.2022.907707 (PMC9501867; doi:10.3389/fnbeh.2022.907707)
Supplement: Supplementary file 3 [file Image_3.pdf]

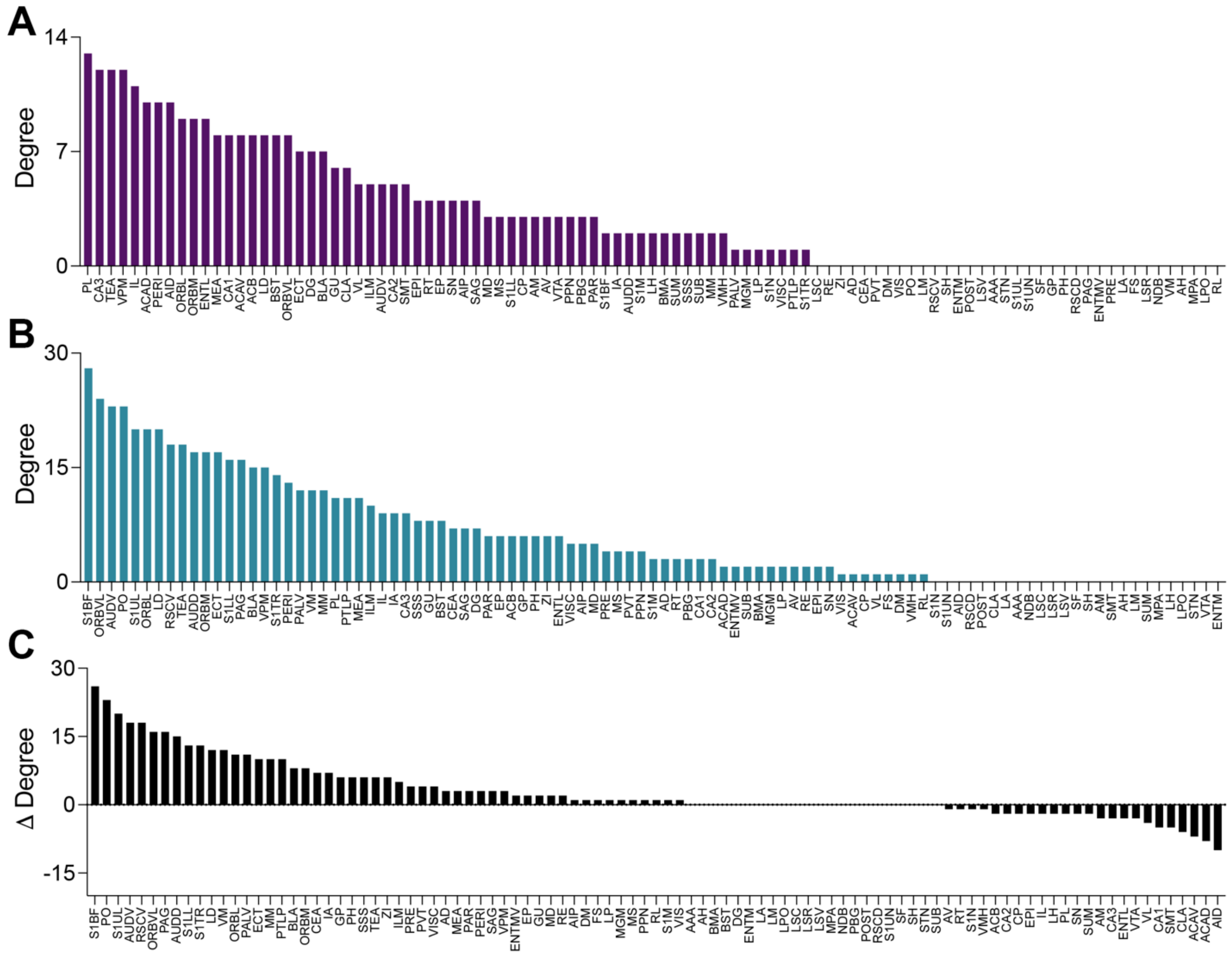

Supplementary Figure S3: Morris water task training alters degree distribution.
The degree (i.e., number of functionally connected regions) of each region from both the (**A**) control and (**B**) MWT trained conditions. (**C**) The relative change in the degree of each region in trained mice compared to control mice.
