# Supplementary material for: Brain-wide neuronal activation and functional connectivity are modulated by prior exposure to repetitive learning episodes
Source: Front Behav Neurosci. 2022 Sep 9;16:907707. doi: 10.3389/fnbeh.2022.907707 (PMC9501867; doi:10.3389/fnbeh.2022.907707)
Supplement: Supplementary file 4 [file Image_4.pdf]

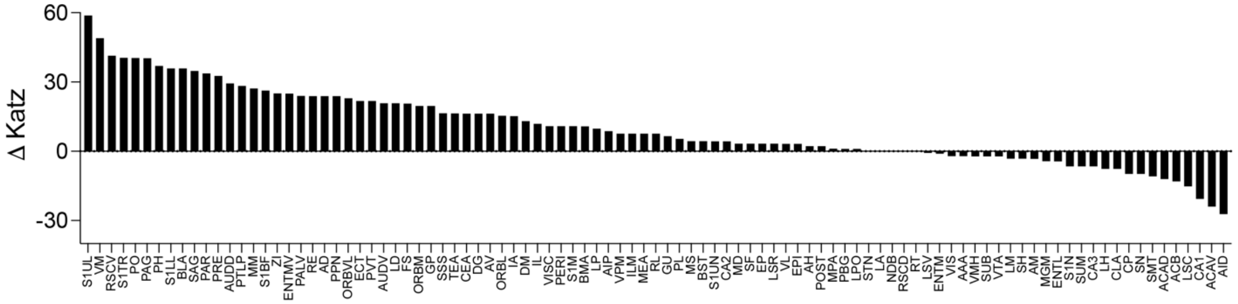

Supplementary Figure S4: Change in Katz centrality. The difference in Katz centrality in Morris water task trained mice relative to untrained controls. Most regions are similar between conditions but there is a small number of regions that show a large increase.
