# Supplementary material for: Brain-wide neuronal activation and functional connectivity are modulated by prior exposure to repetitive learning episodes
Source: Front Behav Neurosci. 2022 Sep 9;16:907707. doi: 10.3389/fnbeh.2022.907707 (PMC9501867; doi:10.3389/fnbeh.2022.907707)
Supplement: Supplementary file 5 [file Table_1.pdf]

Supplementary Table 1: List of brain regions (nodes) in which c-Fos was quantified. Numbers and color coding represent the order and subdivisions in which they appear in the correlation matrices.

#	Abbreviation	Full Name
1	S1bf	Primary somatosensory area, barrel field
2	S1ll	Primary somatosensory area, lower limb
3	S1m	Primary somatosensory area, mouth
4	S1n	Primary somatosensory area, nose
5	S1tr	Primary somatosensory area, trunk
6	S1ul	Primary somatosensory area, upper limb
7	S1un	Primary somatosensory area, unassigned
8	SSs	Supplemental somatosensory area
9	IL	Infralimbic area
10	GU	Gustatory areas
11	VISC	Visceral areas
12	AUDd	Dorsal auditory area
13	AUDv	Ventral auditory area
14	VIS	Visual areas
15	ACAd	Anterior cingulate area, dorsal part
16	ACAv	Anterior cingulate area, ventral part
17	PL	Prelimbic area
18	ORBl	Orbital area, lateral part
19	ORBm	Orbital area, medial part
20	ORBvl	Orbital area, ventrolateral part
21	AId	Agranular insular area, dorsal part
22	AIp	Agranular insular area, posterior part
23	RSCd	Retrosplenial area, dorsal part
24	RSCv	Retrosplenial area, ventral part
25	PTLp	Posterior parietal association areas
26	TEa	Temporal association areas
27	PERI	Perirhinal area
28	ECT	Ectorhinal area
29	CA1	Ammon's horn, CA1 field
30	CA2	Ammon's horn, CA2 field
31	CA3	Ammon's horn, CA3 field
32	DG	Dentate gyrus
33	ENTl	Entorhinal area, lateral part
34	ENTm	Entorhinal area, medial part
35	ENTmv	Entorhinal area, medioventral part
36	PAR	Parsubiculum
37	POST	Postsubiculum
38	PRE	Presubiculum
39	SUB	Subiculum
40	CLA	Claustrum
41	EP	Endopiriform nucleus
42	LA	Lateral amygdalar nucleus
43	BLA	Basolateral amygdalar nucleus
44	BMA	Basomedial amygdalar nucleus
45	AAA	Anterior amygdalar area
46	CEA	Central amygdalar nucleus
47	IA	Intercalated amygdalar nucleus
48	MEA	Medial amygdalar nucleus

#	Abbreviation	Full Name
49	CP	Caudoputamen
50	ACB	Nucleus accumbens
51	FS	Fundus of striatum
52	LSc	Lateral septal nucleus, caudal part
53	LSr	Lateral septal nucleus, rostral part
54	LSv	Lateral septal nucleus, ventral part
55	SF	Septofimbrial nucleus
56	SH	Septohippocampal nucleus
57	GP	Globus pallidus
58	PALv	Pallidum, ventral region
59	MS	Medial septal nucleus
60	NDB	Diagonal band nucleus
61	BST	Bed nucleus of the stria terminalis
62	VL	Ventral lateral nucleus of the thalamus
63	VM	Ventral medial nucleus of the thalamus
64	VPM	Ventral posteromedial nucleus of the thalamus
65	MGM	Medial geniculate complex, medial part
66	LP	Lateral posterior nucleus of the thalamus
67	PO	Posterior complex of the thalamus
68	AV	Anteroventral nucleus of the thalamus
69	AM	Anteromedial nucleus of the thalamus
70	AD	Anterodorsal nucleus of the thalamus
71	LD	Lateral dorsal nucleus of the thalamus
72	MD	Medial dorsal nucleus of the thalamus
73	SMT	Submedial nucleus of the thalamus
74	PVT	Paraventricular nucleus of the thalamus
75	RE	Nucleus of reuniens
76	ILM	Intralaminar nuclei of the dorsal thalamus
77	RT	Reticular nucleus of the thalamus
78	EPI	Epithalamus
79	DM	Dorsomedial nucleus of the hypothalamus
80	AH	Anterior hypothalamic area
81	LM	Lateral mammillary nucleus
82	MM	Medial mammillary nucleus
83	SUM	Supramammillary nucleus
84	MPA	Medial preoptic area
85	VMH	Ventromedial hypothalamic nucleus
86	LH	Lateral hypothalamic nucleus
87	LPO	Lateral preoptic nucleus
88	PH	Posterior hypothalamic nucleus
89	STN	Subthalamic nucleus
90	ZI	Zona incerta
91	PAG	Periaqueductal gray
92	VTA	Ventral tegmental area
93	SN	Substantia nigra
94	PPN	Pedunculopontine nucleus
95	SAG	Nucleus sagulum
96	PBG	Parabigeminal nucleus
97	RL	Rostral linear nucleus raphe
